# Evaluation of the Pharmacological Potential of *Psidium guajava* (Guava) and Its Anticancer Effect

**DOI:** 10.3390/ph19040561

**Published:** 2026-03-31

**Authors:** Mariana Toma, Laura Ancuta Pop, Ioana Berindan-Neagoe, Ancuta Jurj, Lajos Raduly, Dorel Hoza, Vasilica Luchian, Ligia Ion, Radu Burlacu, Floricuta Ranga

**Affiliations:** 1 Fruit Growing Department, University of Agronomic Sciences and Veterinary Medicine, 59 Marasti Avenue, 011464 Bucharest, Romania; mariana.toma@doctorat.usamv.ro (M.T.); dorel.hoza@horticultura-bucuresti.ro (D.H.); vasilica.luchian@horticultura-bucuresti.ro (V.L.); r.burlacu@usamv.ro (R.B.); 2 Genomics Department, MEDFUTURE—Institute for Biomedical Research, “Iuliu Hatieganu” University of Medicine and Pharmacy, No. 23, Gheorghe Marinescu Street, 400337 Cluj-Napoca, Romania; ioana.neagoe@umfcluj.ro (I.B.-N.); anca.jurj@umfcluj.ro (A.J.); lajos.raduly@umfcluj.ro (L.R.); 3 Biochemistry Department, University of Agronomic Sciences and Veterinary Medicine, 3–5 Calea Manastur, 400374 Cluj-Napoca, Romania; florica.ranga@usamvcluj.ro

**Keywords:** apoptosis, autophagy, cancer substitute therapy, cell lines, colon, *Psidium guajava* ethanolic extract, herbal medicine, prostate

## Abstract

**Background**: *Psidium guajava* L. (*Psidium guajava*) is an edible plant; its parts are widely used to cure and prevent many health disorders. *Psidium guajava* leaves contain a wide array of polyphenols that inhibit peroxidation and may play a role in the prevention and treatment of common, degenerative chronic disorders such as diabetes, cardiovascular disease, and cancer. Colon cancer is the third most common type of cancer diagnosed and the second most common cause of cancer-related deaths globally. In contrast, prostate cancer is the second most diagnosed cancer and the fifth leading cause of cancer-related death worldwide. This study aims to evaluate the pharmacological potential of the *Psidium guajava* plants cultivated in Romania on colon and prostate cancer cell lines. **Methods**: Phenolic compounds extraction was made using an average sample of all nine *Psidium guajava* varieties. Analyses were carried out using a HP-1200 liquid chromatograph. The effect of the alcoholic extract of *Psidium guajava* leaves was tested on two colon cancer and one prostate cancer cell line as in vitro models. **Results**: The *Psidium guajava* leaf extract exhibited anticancer activity against the tested cell lines, with decreased proliferation, increased apoptosis, and cell cycle arrest. The extract reduced the cancer cell line’s migration and invasion capacity, demonstrating greater selectivity for the colon cancer cell line than for the prostate cancer cell lines. **Conclusions**: This study provides further proof of the *Psidium guajava* plant’s anticancer properties against colon cancer cell lines. Further studies are needed to confirm its use either alone or in conjunction with conventional cancer treatments as an alternate treatment for certain kinds of malignancies.

## 1. Introduction

*Psidium guajava* (*Psidium guajava*) is native to Central and South America, the West Indies, Mexico, Florida, Louisiana, and Arizona, and is naturalized in parts of Africa, the Indian subcontinent, and on numerous oceanic islands. It exceeds most tropical and sub-tropical fruit trees in adaptability, productivity, and tolerance to mild cold and light frosts, but it can withstand temperatures down to 5 °C. The optimal temperature for summer growth is above 15 °C. These characteristics suggest that *Psidium guajava* should also be acclimatized to other climates, such as Romania’s. Currently, the demands of a hectic lifestyle have increased interest in natural remedies, as synthetic medications can cause numerous adverse effects, as also agreed by the World Health Organization [1]. *Psidium guajava* L. (guava) is an edible plant; its parts are widely used to cure and prevent many health disorders. The scientific evidence of the ethnomedical applications of *P. guajava* began in the 1940s [2]. Therefore, its efficacy and safety have empirically been confirmed [3] by toxicity studies on animals and controlled human studies [4,5]. *Psidium guajava* leaves are a rich source of minerals, such as calcium, potassium, sulphur, sodium, iron, boron, magnesium, manganese, and vitamins C and B, and various bioactive compounds, including tannins, flavonoids, and saponins. The accumulation of plant polyphenols depends on soil/substrate, environmental factors (e.g., temperature, light, and humidity), various stressors, cultivation practices, harvest time, and other factors. Moreover, because the plants are living under stressful conditions, antioxidants accumulate in greater amounts (an adaptation necessary for plant survival). The bioactive compounds from *Psidium guajava* leaf can inhibit the peroxidation reaction [6], playing a role in degenerative chronic disorders, like diabetes [7], cardiovascular disease [8], and cancer [9]. They also show effect against SARS-CoV-2 [10] and other viral infections [11], having antibacterial and anticancer effects [12,13,14,15,16,17,18,19]. The plant can restore the body’s functions during and after chemotherapy or radiotherapy [20]. Nearly 10 million deaths worldwide, or nearly one in six deaths, were caused by cancer in 2020 [1]. Colon cancer is the third most common type of cancer diagnosed and the second most common cause of cancer-related deaths globally [1]. Due to the many advantages in the treatment of colon cancer, most of the patients can benefit from surgical treatment, but still, some patients have recurrent or metastatic disease [21]. Prostate cancer is the second most commonly diagnosed cancer and the fifth leading cause of cancer-related death worldwide [22]. The main treatment option for localized prostate cancer is surgery [23], but there are cases with hormone-refractory prostate adenocarcinoma with metastasis [24]. With all the advances in treatment, anticancer drugs still present adverse effects on normal cells, or patients develop resistance to treatment [25].

This article aims to evaluate the anticancer effects of the ethanolic extract of *Psidium guajava* leaf from plants acclimatized in Romania, which showed some differences in the quantity of bioactive compounds, on colon and prostate cancer cell lines using various functional assays, helping to investigate if this extract could be used as an alternative to conventional therapy, as well as if it could be used as a mixture of bioactive compound, which is easier and cheaper to obtain.

## 2. Results

### 2.1. Biochemical Analysis

The 2021 analysis provided a baseline for the pooled extract used in biological assays, whereas the 2022 data expanded the investigation to variety-specific biochemical profiles.

In 2021, physio and bio-chemical analyses of *Psidium guajava* leaves were performed, as follows: humidity—42.87% fresh leaf, 10.30% dried leaf; total phenolic content—63.48 mg GAE/100 g fresh leaf, 85.48 mg GAE/100 g dried leaf; total flavonoid content—29.59 mg rutin/100 g fresh leaf, 22.46 mg rutin/100 g dried leaf; caffeic acid—0.574 mg /100 g, 0.190 mg/100 g dried leaf. As the results show, after 5 days of natural air-drying, water leaf content decreased by 75.97%, total phenolic content increased by 34.66%, total flavonoid content decreased by 24.10%, and caffeic acid decreased by 66.90%.

In 2021, the highest values (mg/g) were recorded for castalagin (7.414), followed by geraniin (5.959) and guavinoside B (3.551), while in 2022, they were found for catechin (10.85), procyanidin (trimer—11.29; trimer isomer—7.89; dimer—6.49) and guavinoside C (6.25), as are represented in Table 1 and Table 2.

**Table 1:** Identification of *Psidium guajava* leaf phenolic compounds—2021 *.

Rt (min)	UV λmax (nm)	[M + H] + (*m*/*z*)	Phenolic Compounds	mg/g
12.36	280	935	Castalagin	7.414
12.79	270	953	Geraniin	5.959
19.41	263.356	545	Guavinoside B	3.551
3.16	270	139	Hydroxybenzoic acid	2.964
10.41	280	579	Procyanidin dimer isomer	2.48
17.18	258.356	465.303	Quercetin-xyloside	2.386
17.43	259.356	465.303	Quercetin-arabinoside	2.253
11.36	280	579	Procyanidin dimer isomer	2.203
12.61	280	291	Catechin	2.203
16.24	259.355	465.303	Quercetin-galactoside	2.197
8.87	280	595	Prodelphinidin dimer	2.076
9.58	280	307	Gallo-catechin	1.985
16.38	258.355	465.303	Quercetin-glucoside	1.809
14.74	280	579	Procyanidin dimer isomer	1.521
16.93	261.356	481.319	Myricetin-glucoside	1.257
13.97	280	867	Procyanidin trimer	1.216
18.69	265.355	587	Guavinoside C	1.088
21.06	283	695	Guavin B	0.992
21.88	260.355	303	Quercetin	0.921
19.2	265.356	545	Guavinoside A	0.914
15.56	261.356	481.319	Myricetin-galactoside	0.654
13.3	280	937	Casuarictin	0.474
14.95	264.356	451.319	Myricetin-arabinoside	0.377
5.54	280	171	Gallic acid	0.289
Total of analyzed phenolics				49.185

**Figure 1:**
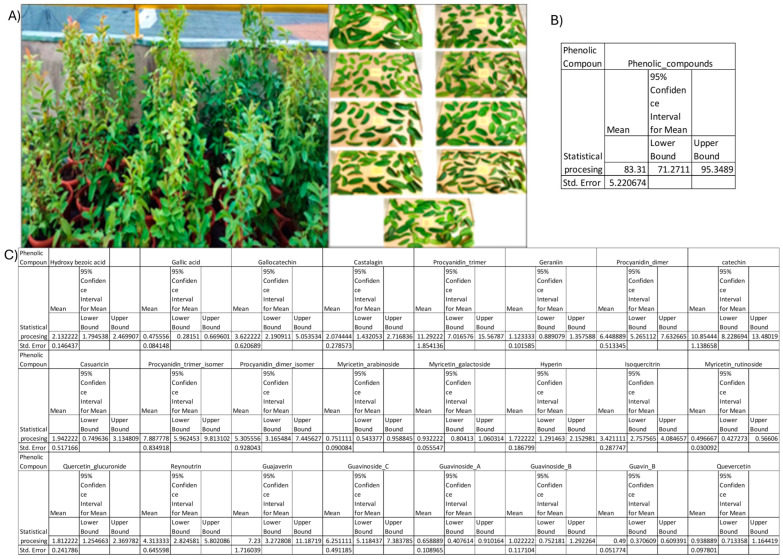
(**A**) Psidium guajav as cultivated at the “Horting” Institute and leaf samples of analyzed varieties. (**B**) confidence interval and standard error of all phenolic compounds identified in the extract (CiM = confidence interval of the mean); (**C**) confidence interval and standard error of the analyzed phenolic compounds (CiM = confidence interval of the mean). Data are presented as mean ± standard error (SE), with the 95% confidence interval (CI) reported for each mean, including lower and upper bounds.

* Mean of nine *Psidium guajava* varieties. Values for standard error (SE) and 95% confidence interval (CI) are presented in Figure 1.

**Table 2:** Evaluation of phenolic compounds found in *Psidium guajava* leaf upon variety—2022.

Rt (min)	UV λmax (nm)	[M + H] + (*m*/*z*)	Compound	Allahabad Sapheda	Bianca	Florida	Florida Tropical	Red Apple	Red Giant	Ruby Supreme	Thai Apple	Thai Ruby	Compound Average of All Varieties
3.16	1.76	139	Hydroxybenzoic acid	1.76	2.13	2.53	2.58	1.59	2.27	2.55	1.42	2.36	2.13
5.24	0.3	171	Gallic acid	0.3	0.33	0.48	0.81	0.17	0.46	0.86	0.21	0.66	0.48
9.48	2.45	307	Gallo-catechin	2.45	1.78	3.51	3.11	1.68	5.94	7.22	3.98	2.93	3.62
10.35	1.36	935	Castalagin	1.36	1.91	3.16	2.83	1.09	2.67	2.82	0.89	1.94	2.07
11.3	5.43	579	Procyanidin dimer	5.43	4.42	8.07	7.24	4.3	8.59	7.09	7.14	5.76	6.49
12.32	11.48	291	Catechin	11.48	11.03	16.1	8.49	6.51	15.8	12.11	8.7	7.54	10.85
12.63	20.43	867	Procyanidin trimer	20.43	7.12	16.9	8.22	3.88	17.3	9.51	10.23	8.05	11.29
13.14	1.18	953	Geraniin	1.18	0.9	1.82	0.98	0.9	1.39	0.1	0.99	0.95	1.02
13.26	0.65	937	Casuarictin	0.65	1.67	4.05	1.57	0.57	5.33	1.6	1.49	0.85	1.98
13.89	11.87	867	Procyanidin trimer isomer	11.87	4.25	9.42	10.4	4.99	6.89	8.79	8.08	6.34	7.89
14.52	7.36	579	Procyanidin dimer isomer	7.36	2.58	6.25	5.55	2.8	11.3	4.99	3.71	3.2	5.31
14.71	0.57	451.32	Myricetin-arabinoside	0.57	0.87	1.23	0.53	0.82	1.86	0.45	0.56	0.64	0.84
15.28	0. 76	481.32	Myricetin-galactoside	0.76	0.84	1.11	0.93	0.92	1.16	0.79	1.14	0.74	0.93
15.58	0.41	627.32	Myricetin-rutinoside	0.41	0.41	0.48	0.6	0.53	0.42	0.53	0.66	0.43	0.5
16.01	0.99	465.3	Quercetin-galactoside (Hyperin)	0.99	2.45	2.09	2.33	1.29	1.51	2.06	1.33	1.91	1.77
16.18	2.51	465.3	Quercetin-glucoside (Isoquercitrin)	2.51	4.41	3.31	4.35	2.66	1.98	3.67	3.93	3.97	3.42
16.6	1.01	479.3	Quercetin-glucuronide	1.01	1.62	2	1.94	1.05	1.25	2.03	3.39	2.02	1.81
16.85	1.97	435.3	Quercetin-xyloside (Reynoutrin)	1.97	4.17	4.59	4.41	2.88	2.17	4.53	8.22	5.88	4.31
17.08	2.37	435.3	Quercetin-arabinoside (Guajaverin)	2.37	5.04	6.98	6.65	3.37	3.24	6.84	18.63	11.95	7.23
18.34	4.16	58	Guavinoside C	4.16	8.48	6.59	6.44	7.12	7.33	6.03	3.82	6.29	6.25
19.07	0.43	545	Guavinoside A	0.43	0.72	0.57	0.64	1.47	0.68	0.59	0.48	0.35	0.66
19.41	0.59	545	Guavinoside B	0.59	1.52	1.24	1.01	0.61	1.05	1.52	0.91	0.75	1.02
20.31	0.27	695	Guavin B	0.27	0.72	0.55	0.38	0.66	0.44	0.4	0.36	0.63	0.49
21.31	0.96	303	Quercetin	0.96	1.06	0.91	1.17	1.38	0.86	1	0.8	0.31	0.94
Total of analyzed phenolics per variety				80.51	70.4	104	83	53.2	102	88.1	90.4	76.5	83.31

**Figure 2:**
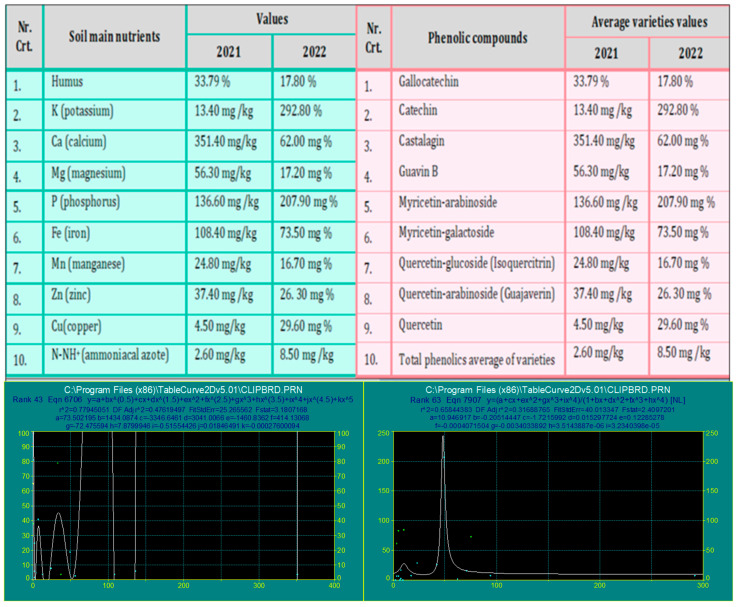
Represents the regression of the main nutrients of soil and the main phenolic compounds of leaves in 2021 and 2022. In 2021, peak values of phenolic compounds were found for castalagin (7.41 mg/g), guajaverin (2.25 mg/g), and catechin (2.20 mg/g), while in 2022, they were found for catechin (10.85 mg/g), myricetin-galactoside (7.71 mg/g), and guajaverin (7.23 mg/g). Multiple linear regression was performed to evaluate the relationships between leaf phenolic compounds and substrate nutrients. Statistical significance of the regression coefficients was assessed using the F-test, with *p* < 0.05 considered significant. In addition, 95% confidence intervals are shown for each regression line.

Values are reported for each phenolic compound for the analyzed varieties. Statistical processing, including regression analysis and 95% confidence intervals for 2021 and 2022, is presented in Figure 2.

### 2.2. Soil and Leaf Compounds Statistics Processing

Regarding the 95% confidence interval (Figure 1), values far from the mean, especially those outside the lower or upper bounds, indicate higher uncertainty in the estimated percentage if the experiments were repeated under the same conditions. Regarding the standard error, lower SE values reflect higher uniformity of the analyzed items and greater precision of the method. The statistical processing is based on the data presented in Table 1. Various studies in the field have shown the correlation between soil nutrients and plant growth, development, and accumulation of bio-compounds in plants [26,27,28,29]. Figure 2 represents the accumulation of phenolic compounds in *Psidium guajava* leaves, according to the main nutrients’ substrate supplied.

Various correlations among the cultivation substrate, growth dynamics, and leaf bio-compounds were statistically analyzed using regression analysis and confidence intervals. The statistical findings exhibit the enormous diversity of the analyzed varieties and the variability of their phenolic compounds.

A judicious application of macro- and micronutrients led to a higher phenolic content in the plant. Mirroring 2021 and 2022, an increase in potassium (195.42%), copper (186.68%), ammonia nitrogen (169.41%), and phosphorus (134.30%) in the substrate led to a higher accumulation of myricetin-arabinoside (194.93%), myricetin-galactoside (191.57%), catechin, (179.72%), quercetin-arabinoside (168.88%), quercetin-glucoside (147.08%) and gallo-catechin (145.00%). The mean of phenolic compounds from the analyzed *Psidium guajava* varieties increased by 169.36%.

### 2.3. Selectivity of Psidium guajava Extract to Cancer Cells

Cell cytotoxicity was measured following exposure to *Psidium guajava* extract to generate dose–response curves and determine the optimal concentrations of *Psidium guajava* extract. The RKO, HCT-116, CCD18Co, 22RV1, and PWR-1E were treated at 24, 48, and 72 h (Supplementary Figure S1). We selected the best results at 24 h, with optimal concentrations of 3.15 mg/mL for HCT-116, 1.575 mg/mL for RKO, and 7.875 mg/mL for CCD18Co, 22RV1, and PWR-1E. To evaluate the selectivity of *Psidium guajava* extract for cancer cells, the selectivity index (SI) must be calculated, defined as the ratio of the cytotoxic concentration for non-cancer cells to that for cancer cells. In this study, it is clear that, for colon cancer cell lines, the concentration required to achieve toxicity is lower (3.15 mg/mL for HCT-116 and 1.575 mg/mL for RKO) than that for a normal cell line (7.875 mg/mL for CCD18Co). However, when it comes to prostate adenocarcinoma, the concentration is the same for both types of cell lines. So, we can say that the *Psidium guajava* extract presents selectivity toward the colon cancer cell lines with 2.5-5-fold higher SI for HCT-116 (SI = 2.5) and RKO (SI = 5) cell lines.

### 2.4. Efficiency of Psidium guajava Extract to Inhibit Colony Formation and Promote Apoptosis

**Figure 3:**
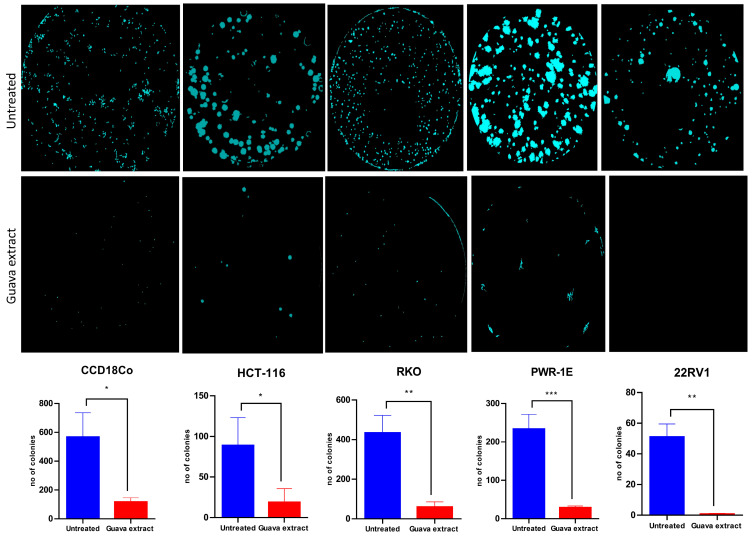
Both cancer and normal colon cell lines were prevented from forming colonies when treated with the following concentrations of *Psidium guajava* extract: 7.875 mg/mL for CCD18Co, 22RV1, and PWR-1E, 3.15 mg/mL for HCT-116, and 1.575 mg/mL for the RKO cell line for 24 h treatment. The colony formation assay images for each treatment are displayed, along with the number of colonies (data are shown as mean ± SD (n = 3 replicates); statistical significance was determined using a two-sided unpaired *t*-test, significance levels, * = 0.0343 for HCT116, * = 0.0105 for CCD18Co, ** = 0.021 for RKO, *** = 0.0008 for PWR-1E, and ** = 0.015 for 22RV1).

**Figure 4:**
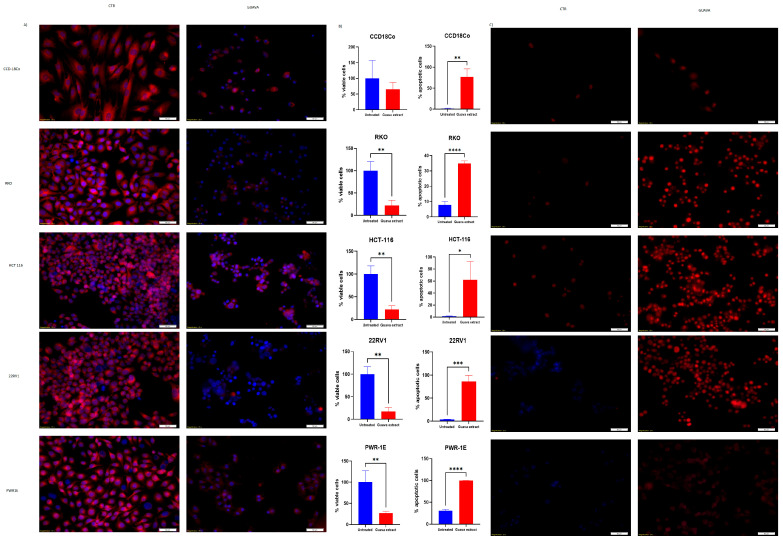
Apoptosis and autophagy were assessed using fluorescence microscopy after exposing CCD18Co, RKO, HCT-116, PWR-1E, and 22RV1 cell lines to *Psidium guajava* with the following concentrations: 7.875 mg/mL for CCD18Co, 22RV1, and PWR-1E, 3.15 mg/mL for HCT-116, and 1.575 mg/mL for the RKO cell line for 24 h treatment (20× magnification). (**A**,**B**) The results indicate a notable reduction in the number of treated cells in both colon and prostate cancer cell lines, as well as in normal cell lines, compared with the control group after 24 h of *Psidium guajava* extract treatment. Additionally, treated cells exhibited a compromised mitochondrial membrane potential relative to the control group, suggesting the occurrence of apoptosis (data expressed as mean ± SD (n = 3 replicates). Statistical significance was determined using a two-sided unpaired *t*-test. Significance levels; viable cells: *p* ** = 0.0045 for RKO, *p* ** = 0.0062 for HCT-116; apoptotic cells: *p* ** = 0.0025 for CCD18Co, *p* **** < 0.0001 for RKO, *p* * = 0.0329 for HCT-116, *p* ** = 0.0098 for PWR-1E, and *p* ** = 0.0016 for 22RV1; apoptotic cells: *p* *** = 0.0004 for 22RV1 and *p* **** < 0.0001 for PWR-1E). (**C**) Following 24 h of *Psidium guajava* extract treatment, blue fluorescence indicates MDC (Monodansylcadaverine), while red fluorescence represents propidium iodide (PI). An increased presence of MDC-labeled vesicles was observed in treated cells compared with controls in both prostate and colon cancer cell lines. The impact of *Psidium guajava* extract on prostate cancer and normal cell lines is presented (data are shown as mean ± SD (n = 3 replicates). Statistical significance was determined using a two-sided unpaired *t*-test) (CTR = control, untreated cells).

Next, the effect of *Psidium guajava* extract on colony formation in the tested cell lines was investigated. According to the findings, *Psidium guajava* extract at the aforementioned concentration inhibits colony formation relative to the control group across all tested cell lines (Figure 3). *Psidium guajava* extract therapy promotes apoptosis and autophagy in cell lines from prostate and colon cancer. We conducted a functional analysis of cancer progression relative to the control group using CCD18Co, HCT-116, RKO, 22RV1, and PWR1E cell lines. Twenty-four hours after treatment, apoptosis was assessed by fluorescence microscopy following staining with the TMRE and Hoechst dyes. The fluorescent dyes, TMRE and Hoechst, are markers for mitochondria and nuclei, respectively. In Figure 4A, we observed that cells in the control group stained with TMRE present an undisturbed mitochondrial membrane potential. Thus, this aspect demonstrated that the cells were unaffected by stimuli that could alter their morphology or trigger cell death. Meanwhile, the effect of *Psidium guajava* extract on HCT-116, RKO, and 22RV1 cells was significantly different from that of the control group. Apoptotic reactions have been strongly expressed regarding the colon and prostate cancer cell lines. This therapy disrupts cell membrane asymmetry, induces cell shrinkage, and promotes nuclear fragmentation, thereby triggering cell death. Nucleus fragmentation was highlighted through Hoechst staining. It was observed that the effect of this treatment on cells was markedly increased in the presence of fragmented and polynuclear cells, compared with the untreated group, leading to apoptosis, as determined by TMRE-Hoechst staining. Additionally, we performed a statistical analysis of viable HCT-116, RKO, and 22RV1 cells to determine whether the initiation of apoptosis is associated with a reduction in cell numbers. *Psidium guajava* extract treatment reduced cell viability, indicating a significant effect on the tested cancer cell lines. In Figure 4A, the second graphic on the right side shows the results of apoptosis evaluation in prostate and colon cancer cells and normal cells. In this experiment, we evaluated the percentage of cells positive for Cas3/Cas7, which are markers of apoptosis. As we can see, the apoptotic cells are increased in *Psidium guajava* extract-treated cells compared to untreated cells.

Furthermore, we sought to determine whether the aforementioned therapy induces apoptosis and whether the decrease in cell viability is directly attributable to it. Autophagy activity was assessed by PI and MDC staining and visualized using a fluorescence microscope.

The MDC dye is a marker for autophagic vacuoles, which appear as dot-like structures in the cytosol, while the PI dye is used to identify apoptotic and necrotic cells.

As shown in Figure 4B, we observed only apoptotic cells in *Psidium guajava* extract-treated cells compared to control cells, indicating that the cancerous cells were not activated. This effect was observed to a lesser extent in the normal cell lines.

### 2.5. Cellular Morphology and Cytoskeletal Evaluation

**Figure 5:**
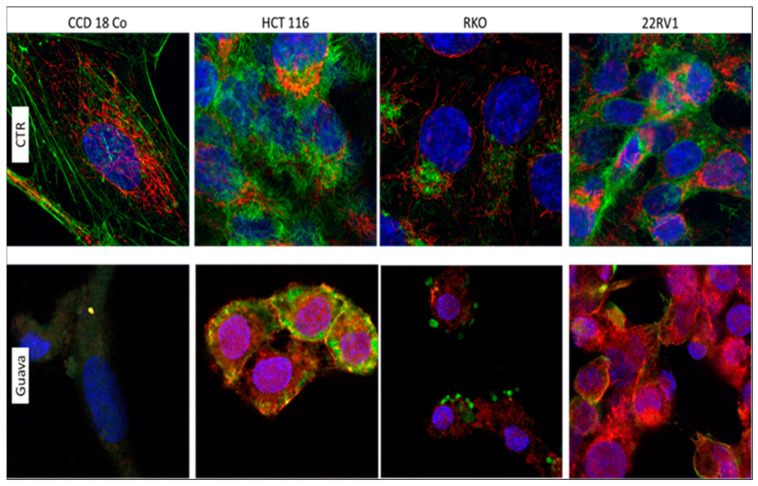
Modifications in the cytoskeleton following treatment with *Psidium guajava* extract at the concentrations 7.875 mg/mL for CCD18Co, 22RV1, and PWR-1E, 3.15 mg/mL for HCT-116, and 1.575 mg/mL for RKO, 24 h after treatment using confocal microscopy and cytoskeleton staining by Phalloidin-FITC (Fluoresceina izotiocianat) (green), nucleus staining by DAPI (4′,6-diamidino-2-phenylindole) (blue), and mitochondria staining by MytoTracker (red). Photographs were taken with a 60× oil-immersed objective. (CTR = control, untreated cells).

The morphological response to *Psidium guajava* extract treatment was evaluated by confocal microscopy to assess cellular morphology. Confocal microscopy provides more discrete features, such as changes in the cytoskeleton, consequently affecting the status of actin and tubulin within a cell. Actin filaments significantly facilitate cell infiltration. As shown in Figure 5, actin rearrangement was stained with Phalloidin-FITC dye, an agent involved in actin filaments stabilization and depolymerization prevention, and cell nuclei were identified by DAPI staining and mitochondria by MytoTracker. Actin-driven membrane disruption and fragmented nuclei were observed in treated cells. The response of cancer cells to therapy was significantly higher than that of the untreated group, which exhibited cellular morphology with notable alterations and a moderately extended contour. Overall, the cytoplasmic zone is diminished after therapy, and large nuclei were also observed as a stress response, without signs of fragmentation. Meanwhile, we observed increased mitochondrial activity in HCT-116 and 22RV1 cell lines, whereas mitochondrial activity was reduced in CCD18Co and RKO cell lines. Overall, the therapy affects cytoskeletal integrity and results in irregular or fragmented nuclei compared with the untreated group.

### 2.6. Cell Cycle Assessment of Psidium guajava Extract Treatment

**Figure 6:**
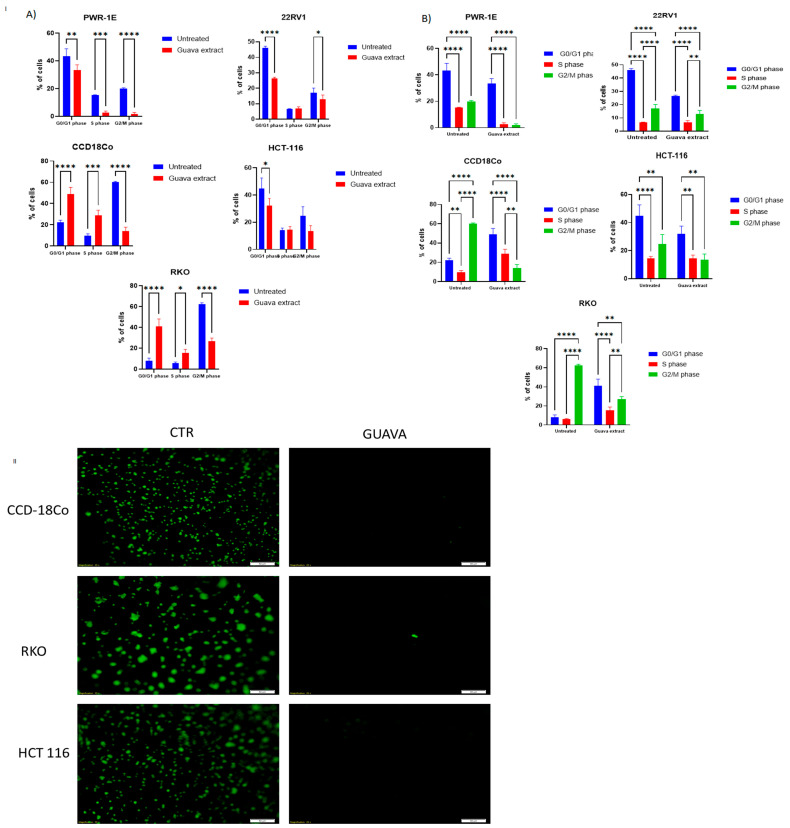
(**I**) Assessment of the impact of *Psidium guajava* extract treatment at the concentrations of 7.875 mg/mL for CCD18Co, 22RV1, and PWR-1E, 3.15 mg/mL for HCT-116, and 1.575 mg/mL for RKO on the tested cell line’s cell cycle stages. (**A**) Bar graph showing cell cycle stages on the X axis; (**B**) bar graph showing untreated and treated cells on the X axis; data are shown as mean ± SD (n = 3 replicates). Statistical significance was determined using two-way ANOVA. Significance levels are indicated as follows: * *p* < 0.05, ** *p* < 0.01, *** *p* = 0.0004, and **** *p* < 0.0001. (**II**) Assessment of colon cancer and normal cell lines’ capacity to invade following *Psidium guajava* extract treatment at 7.875 mg/mL for CCD18Co, 3.15 mg/mL for HCT-116, and 1.575 mg/mL for RKO. (CTR = control, untreated cells).

Uncontrolled growth and proliferation are hallmarks of cancer cells; therefore, it is critical to identify agents that can disrupt these processes. For cell cycle arrest investigation, we used the Celigo Image cytometer for prostate and colon cancer cell lines and normal colon and prostate cell lines after treatment with *Psidium guajava* leaf extract, as described in Figure 6I(A,B). Examining the cell cycle of cancer cells is one method for assessing how a treatment affects cell proliferation. In our instance, we can see that normal prostate cancer cell lines exhibit a significant reduction in G0/G1 and G2/M phases, and a smaller reduction in S-phase populations, indicating an overall suppression of proliferation, rather than cell cycle arrest. The prostate cancer cell lines show an increase in G0/G1 and a decrease in G2/M population, describing a clear G0/G1 cell cycle arrest. The normal colon cell line shows a significant increase in G03G1 and S-phase cell populations and a decrease in G2/M, indicating reduced progression into mitosis. Treatment of the colon cancer cell line HCT-116 reduces cells in the G0/G1 and G2/M phases, with a small change in the S-phase population, indicating a single-phase arrest. After treatment, the RKO cell lines show an increase in the G0/G1 phase and a reduction in the G2/M phase population, with a moderate increase in the S-phase, resulting in a G0/G1 phase arrest. 

### 2.7. Evaluation of Psidium guajava Extracts‘ Effect on the Invasion Ability of Colon Cancer and Normal Cell Lines

The treated and untreated cells were analyzed using Matrigel and Calcein AM staining, and we observed that, in the untreated cells, cells passed through Matrigel. In contrast, treatment significantly affected the ability of both cell types to invade (Figure 6II).

### 2.8. Evaluation of Cell Migration

The wound healing assay, conducted on colon and prostate cancer cell lines, as well as normal cell lines, was assessed at various time points (4 h, 6 h, 8 h, and 24 h) following treatment. Regarding the migration potential of all tested cell lines, we observed that both colon and prostate cancer cell lines exhibited low migration rates, even in the absence of treatment. We evaluated several time intervals (4 h, 6 h, 8 h, and 24 h) for this assay and determined that 24 h was the most appropriate. At this interval, we observe that untreated normal cell lines close the gap, whereas cells treated with *Psidium guajava* extract do not. Regarding the cancer cell lines, we observed inhibited wound healing, and the effects were slightly different compared to the untreated group after 24 h for colon and prostate cancer cell lines (Table 3). These data suggest that the treated cells are less prone to invasion and metastasis.

## 3. Discussion

Bioactive compounds with antioxidant activity in *Psidium guajava* leaves, analyzed in 2021 and 2022, showed values comparable to those reported in the literature on the subject, such as Alam et al. [30] and Zhou et al. [31], highlighting a powerful potential to capture free radicals, but also antibacterial and anti-inflammatory properties [32,33,34].

Due to their effects on the gut and the host microbiome, plant polyphenols are highly valuable for human health. When ingested, the polyphenols reach the colon, where the resident microorganisms can metabolize them, and more than 90% of them are not absorbed [35]. Quercetin, one of the phenolic chemicals examined, is a powerful ally in the fight against diabetes [36], hypertension [37], and cancer [38]. It also supports liver function and general metabolism [39].

Our investigation identified significant concentrations of quercetin, rutin, castalagin, gallo-catechins, and myricetin, among other phenolic compounds well-documented for their potent anticancer effects.

Rutin reduces the effects of oxidized LDL cholesterol and may, therefore, reduce the risk of heart disease. It exhibits anticancer, antioxidant, antiaging, anti-inflammatory, antidiabetic, antihypertensive, antidepressant, antigout, and antimicrobial properties. Caffeic acid exhibits many health benefits, including anticancer, anti-inflammatory, and antiviral properties.

The usefulness of rutin and quercetin in treating influenza infections was demonstrated by Savov et al. [40]. The bioactive compounds identified in the Romanian-acclimatized *Psidium guajava* leaves, particularly quercetin, rutin, and castalagin, are well-documented for their potent anticancer properties. In the present study, the high concentration of these polyphenols correlates with the observed reduction in cell viability and induction of programmed cell death in colon and prostate cancer cell lines. Quercetin, a major component of our extract, has been shown to target mitochondria and disrupt anti-apoptotic pathways, which aligns with our findings of compromised mitochondrial membrane potential and increased Caspase 3/7 activity [41,42]. Similarly, castalagin has been reported to exhibit antiproliferative effects by modulating cellular signaling [43], while catechins and gallo-catechins are known to inhibit angiogenesis and cancer cell progression [44]. Our experimental data demonstrate that the guava extract not only reduces proliferation but also induces a clear G0/G1 or G2/M phase arrest, depending on the cell line. This is consistent with the presence of rhamnoallosan and other phenolic compounds that interfere with the cell cycle machinery [14,45]. Furthermore, the significant inhibition of migration and invasion observed in our functional assays suggests that the complex mixture of bioactive compounds in the Romanian-acclimatized guava leaf extract may reduce the metastatic potential of colorectal and prostate malignancies in vitro.

*Psidium guajava* leaves have attracted the attention of leading research groups and are recognized as a potent ally in cancer therapy. Recent studies have proven the indisputable effects of *Psidium guajava* extracts against various types of malignancies, especially cancer affecting soft tissues, such as colon cancer, prostatic adenocarcinoma [46], invasive ductal carcinoma, and bronchogenic carcinoma.

In the present research, *Psidium guajava* extract was found to have 2.5-5-fold higher selectivity for colon cancer cells than normal cell lines and to inhibit the growth of colon and prostate cancer cell lines. *Psidium guajava* leaf extract contains a novel rhamnoallosan compound with strong anticancer potential, as shown in a prior study [14], which reduced the proliferation of two prostate cancer cell lines, Du145 and LNCaP. On HCT-116, two substances from *Psidium guajava* leaf extract were found to exhibit antiproliferative activity [45]. Additionally, the ethanolic extract of *Psidium guajava* leaves was used to probe cancer cell lines in both in vitro and in vivo investigations [12].

The cell cycle was also affected by *Psidium guajava* treatment in the tested cells in a cell line-dependent manner. A clear G0/G1 phase arrest was observed in the RKO and 22RV1 cell lines, whereas HCT-116 showed broader cell cycle disruption. The normal cell lines tested exhibit altered distribution patterns but no phase-specific arrests, unlike cancer cell lines. The same alteration of proliferation and cell cycle arrest was also observed by Chen et al. in the LNCaP cell line [47].

The current study’s extract was likewise found not to affect normal cell lines but to significantly limit colony formation and induce apoptosis in colon and prostate cancer cell lines. This result is in keeping with recent research on colon and prostate cancer cell lines that demonstrated that components of *Psidium guajava* leaf extract have a potent apoptotic impact and raise the expression of essential apoptotic genes, including p53, p-Junk, Cas8, BCL-2, and Cas9 [48].

## 4. Materials and Methods

### 4.1. Soil Determination of Containerized Psidium guajava

The soil was pretreated and air-dried at 40 °C in accordance with ISO 11464:2006. Macro- and micronutrients were determined by optical emission spectrometry using inductively coupled plasma and by a colorimetric method of discontinuous flow analysis. K, Ca, Mg, B, and N-NH+ were determined in the aqueous extract prepared by mixing soil with ultrapure water at a 1:5 ratio. Fe, Mn, Zn, and Cu were extracted using a buffered DTPA solution in accordance with SR ISO 14870:2002. P was extracted in a 0.5 mol/L sodium bicarbonate solution at a 1:20 ratio, in accordance with SR ISO 11263:1998. Humus content was determined indirectly, depending on the organic carbon content, by multiplying it by a coefficient of 1.7241 (correction factor in relation to the percentage of carbon recovery).

### 4.2. Biochemical Analysis of Psidium guajava Leaves

The *Psidium guajava* plants used in this study belong to nine varieties, namely: *Allahabad Sapheda*, *Bianca*, *Florida*, *Florida Tropical*, *Red Apple*, *Red Giant*, *Ruby Supreme*, *Thai Apple*, and *Thai Ruby*. The *Psidium guajava* plants analyzed in this study were produced by sowing in 2018 (June), 2019 (January and March), and 2020 (June); the seeds were purchased from Live Seeds Ltd., U.K., Feltham (Florida Tropical, Ruby Supreme and Thai Apple varieties), Squeaky Buffalos Tropical Seeds, U.K. (Thai Ruby), Din-is-good soil, Thailand (Red Apple variety), Vivai Mola Della Abbadia SRL, Italy, Naples (Bianca variety), and All U need is Seed, Australia (Florida and Red Giant varieties). The seeds for the production of guava plants in 2020 were collected from a family garden located in India, Uttarakhand State, Roorkee (Allahabad Sapheda variety). The voucher specimen for all *Psidium guajava* varieties analyzed in this study were deposited in 2023 at the Herbarium of the Faculty of Horticulture, within the University of Agronomic Sciences and Veterinary Medicine of Bucharest, under the supervision of the Department of Botany, getting the Accession Numbers in the Official Botanical Collection, as follows: 4084—Allahabad Sapheda, 4085—Bianca, 4086—Florida, 4087—Florida Tropical, 4088—Red Apple, 4089—Red Giant, 4090—Ruby Supreme, 4091—Thai Apple, and 4092—Thai Ruby.

The plants were produced by germination (by seed) at the “Horting” Institute between 2017 and 2022 and cultivated in containers (semi-protected cultivation). From May to October, they remained outdoors (on the institute’s terrace), and in the cold months, they were brought indoors. Biochemical profiling was conducted in two stages: a preliminary assessment of a representative pooled sample in 2021, followed by a comprehensive variety-specific analysis in 2022 to evaluate intra-species variability. Phenolic compounds extraction was conducted using one average sample of all nine *Psidium guajava* varieties, as follows: 1 g dried and crushed *Psidium guajava* sample was extracted with 5 mL ethanol/water 40/60 (*v*/*v*), by vortexing for 1 min using a Heidolph Reax top vortex, sonication for 30 min in an Elmasonic E 15 H sonicator bath, centrifugation at 8000 rpm for 10 min and T = 200 C in an Eppendorf AG 5804 centrifuge. The supernatant was collected, and the above operations were repeated 3 times. The extract was filtered through a Chromafil Xtra nylon 0.45 µm filter, and 20 µL was injected into the HPLC system. An HP-1200 liquid chromatograph with a quaternary pump, autosampler, DAD detector, and MS-6110 single-quadrupole API-electrospray detector (Agilent Technologies, Santa Clara, CA, USA) was used to perform HPLC/DAD/ESI+ studies. Different fragments in the 50–100 eV range were used to identify phenolic compounds in the positive ionization mode. The column was a Phenomenex Kinetex XB-C18 (5 μm; 4.5 × 150 mm). Water that had been acidified by 0.1% acetic acid and acetonitrile that had been acidified by 0.1% acetic acid made up the mobile phase. A multistep linear gradient was delivered, starting with 5% for two minutes, then increasing to 90% over twenty minutes, holding at 90% for four minutes, and finally decreasing to 5% over six minutes. With a flow rate of 0.5 mL/min and an oven temperature of 25 ± 0.5 °C, the analysis took 30 min in total. The scan mode was used to detect positively charged ions via mass spectrometry. The following experimental parameters were used: capillary voltage 3000 V, fragmentor 100 eV, nebuliser pressure 35 psi, nitrogen flow 7 L/min, gas temperature 350 °C, and *m*/*z* 120–1400. Chromatograms were acquired at wavelengths of λ = 280, 340, and 520 nm. Agilent ChemStation software (B.02.01 SR2) was used to acquire the data. Acetonitrile, an HPLC gradient supplied by Merck (Darmstadt, Germany), and water filtered using a direct-Q UV system from Millipore (Burlington, MA, USA) were the chemical reagents and materials utilized for the assessment of phenolic compounds. Sigma (Milwaukee, WI, USA) supplied the pure standards of rutin, cyanidin, catechin, and chlorogenic and gallic acids (>99% HPLC purity). For the quantification of flavanols, a calibration curve was made with catechin (R^2^ = 0.9994), for the quantification of hydroxybenzoic acids, a calibration curve was made with gallic acid (R^2^ = 0.9995), and for hydroxycinnamic acids, a calibration curve was made with chlorogenic acid (R^2^ = 0.9937). Flavanols were quantified as rutin equivalent (R^2^ = 0.9961), and anthocyanins as cyanidin equivalent (R^2^ = 0.9951).

### 4.3. Cell Culture

Two colon cancer cell lines, HCT-116 and RKO, one prostate cancer cell line, 22RV1, one normal colon cell line, CCD18Co, and one normal prostate cell line, PWR-1E, were used in this investigation. The American Type Culture Collection, or ATCC, is the source of all cell lines. First, 10% fetal bovine serum (FBS-Gibco, Waltham, MA, USA) was added to McCoy medium (Gibco, Waltham, MA, USA) for the cultivation of the HCT-116 and RKO cell lines. The CCD18Co cell line was cultivated in MEM medium (Gibco, Waltham, MA, USA) supplemented with 2 mM L-glutamine (Gibco^®^) and 10% FBS (Gibco, Waltham, MA, USA). The PWR-1E cell line was cultivated in Keratinocyte-SFM (Gibco, Waltham, MA, USA) supplemented with Bovine Pituitary Extract (BPE), EGF, Human Recombinant, and 1% Penicillin–Streptomycin (Gibco, Waltham, MA, USA), while the 22RV1 cell line was cultivated in RPMI-1640 (RPMI-1640 Gibco, Waltham, MA, USA), supplemented with 10% FBS (Gibco, Waltham, MA, USA), 2 mM L-glutamine (Gibco^®^), and 1% sodium pyruvate (Gibco, Waltham, MA, USA).Cells kept at 37 °C in a humidified environment with 95% air and 5% CO_2_). To all cell culture media used for the in vitro experiments, we added 0.5 mL of ZellShield (Minerva Biolabs, Berlin, Germany) to 50 mL of medium.

### 4.4. Cytotoxicity Evaluation Using the Celigo Image Cytometer

Using the Celigo Image Cytometer (Nexcelon, Lawrence, MA, USA), confluence analysis was used to assess cell cytotoxicity. After being grown at sub-confluence, the investigated cell lines underwent two rounds of washing in phosphate-buffered saline (PBS 1X). Trypsinization was followed by resuspension in complete culture medium, cell counting, and seeding for the experiment. *Psidium guajava* extract (3.15 g of dried leaves in 40 mL EtOH 40%, corresponding to a concentration of 78.75 mg dried leaf equivalent per ml of extract) was added to cells at a seeding density of 10^4^ cells per well in a 96-well plate. The extract was diluted to 7.875, 3.15, 1.575, and 1.05 mg/mL in cell culture media specific to each cell line. Tests were conducted at 24, 48, and 72 h after treatment, and the data were used to assess the cytotoxic effect of the *Psidium guajava* extract using the Celigo Image Cytometer (Nexcelon, Lawrence, MA, USA).

### 4.5. Apoptosis and Autophagic Vacuoles Assessment Through Fluorescence Microscopy

Using the Multi-Parameter Apoptosis Assay Kit (Cayman cat. no. 600330, Tallin, Estonia) and the Autophagy/Cytotoxicity Dual Staining Kit (Cayman cat. no. 600140,Tallin, Estonia), respectively, apoptotic and autophagic effects were assessed on an Olympus IX71 inver ted microscope according to the manufacturer’s instructions. In 8-well chamber slides, cells were cultivated at a seeding density of 1.2 × 10^4^ cells. Cells were cultured for an additional 24 h at 37 °C after being treated with mass concentrations of *Psidium guajava* extract of 7.875 mg/mL for CCD18Co, 22RV1, and PWR-1E, 3.15 mg/mL for HCT-116, and 1.575 mg/mL for the RKO cell line. The cell’s nucleus was stained with Hoechst dye (20 mM) and examined under a UV lamp to assess apoptosis. The mitochondrial membrane activity potential was assessed at 560/595 nm using TMRE dye (Tetramethyl Rhodamine Ethyl Ester) labeling. Monodansyl cadaverine (MDC) dye was used to stain the autophagic vacuoles, which were then visible under a UV lamp. At 493/636 nm, cellular death was measured with propidium iodide (PI) dye.

### 4.6. Scratch Assay/Wound Healing Assay

Cells were seeded at 2 × 10^5^ per well in a 6-well plate and incubated for 24 h at 37 °C to assess cell migration using the wound healing assay. The cells were incubated at 37 °C for 24 h after being treated with the aforementioned dosages. A 20 μL tip was used to create the wound, and culture medium was then added. The Celigo Image Cytometer (Nexcelom, Lawrence, MA, USA) was used to visualize the wounds at various time points.

### 4.7. Colony Assay

The ability of cancer cells to survive treatment, or the efficacy of cytotoxic drugs, was assessed using a clonogenic assay. After being seeded at a density of 1 × 10^5^ cells per well in a 12-well plate, the cells were allowed to adhere for 24 h, treated with *Psidium guajava* extract concentration, and then incubated for an additional 24 h at 37 °C. Following two PBS washes and a count, 500 cells/well of the investigated cell lines were cultivated in a 6-well plate with complete culture medium. After six to eight days, adherent cells were washed with PBS 1X, the culture media were disposed of, and the plate was examined using the Celigo Image Cytometer (Nexcelon, Lawrence, MA, USA). Crystal violet was used to stain the cells after fixation in 80% methanol. The Celigo tool was used to visualize the plate after staining. The number of *Psidium guajava* extract-treated colonies compared to the number of control colonies generated was used to determine the outcome of the clonogenic assay.

### 4.8. Cell Cycle and Evaluation Using the Celigo Image Cytometer

Cells were seeded at 1.5 × 10^4^ per well in a 96-well plate and cultured for 24 h at 37 °C to assess cell cycle progression. Cells were fixed in 70% ethanol at 4 °C for 30 min after being washed with cold 1× PBS 24 h earlier and treated with *Psidium guajava* extract. After being resuspended in RNase buffer containing 40 μg/mL RNase and 1 µg/mL PI (propidium iodide dye), fixed cells were incubated at 37 °C for 45 min. Following incubation, the plate was washed with 1× PBS and photographed using the Celigo Image Cytometer (Nexcelom, Lawrence, MA, USA).

### 4.9. Apoptosis Evaluation Using the Celigo Image Cytometer

Cells were seeded at 1.5 × 10^4^ per well in a 96-well plate and cultured for 24 h at 37 °C to assess apoptosis. The plate was incubated for 30 min at 37 °C after 100 µL of a solution containing Caspase 3/7 (4 µM) and Hoechst (8 µM) was added to the cells 24 h after the *Psidium guajava* extract treatment. Following incubation, the plate was washed with 1× PBS and photographed using the Celigo Image Cytometer (Nexcelom, Lawrence, MA, USA).

### 4.10. Invasion Assay

For the colon cancer and normal cell lines, we also evaluated the invasion potential of the treated cells using the Corning Matrigel Basement Membrane Matrix according to the manufacturer’s protocol. To visualize the invasive cells, we used Calcein AM staining (Invitrogen, Carlsbad, CA, USA). The cells were then imaged using an Olympus IX71 inverted microscope at 20× magnification.

### 4.11. Cellular Morphology and Cytoskeletal Evaluation

Cells were cultivated on 8-well chamber slides, labeled with Invitrogen’s MitoTracker Mitochondria, incubated for 1 h, and then rinsed with 1× PBS (3×) before fixation for 15 min at room temperature in 4% (*w*/*v*) paraformaldehyde. Following three PBS washes, they were permeabilized for 10 min at room temperature with 0.1% Triton X-100, and then underwent one final wash. Next, we used DAPI and Phalloidin to stain the cells. The slide was imaged at 60× magnification using a Zeiss LSM 900 confocal microscope (Zeiss, Oberkochen, Germany).

### 4.12. Statistical Analysis

The data were analyzed using two-way ANOVA. Regression analysis was used to estimate the regression function, standard error, and confidence intervals. Statistical significance was assessed using Student’s *t*-test and the *F*-test. A significance level of *p* < 0.05 was considered statistically significant. All analyses were performed using Quattro Pro X7 and SPSS software (version 26.0, IBM Corp., Armonk, NY, USA).

## 5. Conclusions

In a prostate cell line, *Psidium guajava* leaf extract exhibited the highest activity at 7.875 mg/mL, whereas in colon cancer, the concentrations were lower: 3.15 mg/mL and 1.575 mg/mL. Furthermore, we observed significant morphological modifications after treatment with *Psidium guajava* extract. We observed that the cytoskeleton of cancer cells was severely affected by rearrangements of actin filaments, membrane disruption, and fragmented nuclei. Additionally, mitochondrial activity was increased in HCT-116 cells and decreased in RKO cells, as shown in Figure 4. *Psidium guajava* extract has also shown inhibition of the migration activity of colon and prostate cancer cell lines.

Even though both cancer types present modifications after *Psidium guajava* extract treatment, we observed a stronger selectivity toward colon cancer cell lines than toward prostate cancer cell lines.

In conclusion, the ethanolic extract of Romanian-acclimatized *Psidium guajava* leaves demonstrated significant in vitro anticancer efficacy against prostate and colon cancer cell lines. The extract effectively induced apoptosis, promoted cell cycle arrest, and inhibited the migration and invasion capacities of the tested cancer cells, while exhibiting a high selectivity index compared to normal cell lines. These findings establish a clear relationship between the soil nutrient profile and the resulting pharmacological potential of the plant. While these results are promising, further in vivo studies and clinical evaluations are necessary to confirm the safety and therapeutic potential of this extract as a complementary treatment for certain malignancies.

Against the most terrible disease of the 21st century—cancer, *Psidium guajava* extract may be a possible cure that exerts cytoprotective effects and supports normal cellular functions. The highly valuable yet underexplored pharmacological potential of *Psidium guajava* plants may hold the key to treating many of the ailments facing modern society.

## Figures and Tables

**Table 3:** Confluence % measured by the Celigo Image Cytometer for all the cell lines at all time intervals.

Cell Line	Time				
	0 h	4 h	6 h	8 h	24 h
CCD18 Co Control	40.42%	50.35%	67.89%	72.55%	99.91%
CCD18 Co *Psidium guajava* extract	68.01%	70.05%	80.52%	82.78%	98.63%
HCT-116 Control	79.34%	80.05%	82.59%	94.90%	95.95%
HCT-116 *Psidium guajava* extract	72.86%	72.90%	71.95%	72.05%	75.83%
RKO Control	59.53%	59.51%	65.93%	79.88%	80.70%
RKO *Psidium guajava* extract	62.21%	62.00%	61.98%	62.25%	61.54%
PWR-1E Control	21.05%	48.88%	59.75%	70.85%	84.05%
PWR-1E *Psidium guajava* extract	66.20%	69.05%	72.85%	73.95%	87.75%
22RV1 Control	72.15%	75.15%	78.09%	81.05%	82.29%
22RV1 *Psidium guajava* extract	66.16%	67.49%	68.59%	70.45%	72.01%

## Data Availability

The original contributions presented in this study are included in the article/Supplementary Materials. Further inquiries can be directed to the corresponding authors.

## Supplementary Materials

The following supporting information can be downloaded at: https://www.mdpi.com/article/10.3390/ph19040561/s1, Figure S1: The anti-proliferative effect of *Psidium guajava* measured at (A) 24H after treatment with 78.75, 7.875, 3.15, 1.575, and 1.05 mg/mL, (B) 48H after treatment with 78.75, 7.875, 3.15, 1.575, and 1.05 mg/mL, and (C) 72H after treatment with 78.75, 7.875, 3.15, 1.575, and 1.05 mg/mL. Log(conc, mg/mL) = log(concentration of bioactive compounds, mg/mL) (mean ± SD, n = 3).
