# Supplementary material for: Evaluation of the Pharmacological Potential of Psidium guajava (Guava) and Its Anticancer Effect
Source: Pharmaceuticals (Basel). 2026 Mar 31;19(4):561. doi: 10.3390/ph19040561 (PMC13118898; doi:10.3390/ph19040561)
Supplement: Supplementary file 1 [file pharmaceuticals-19-00561-s001.zip › pharmaceuticals-4135062-supplementary.pdf]

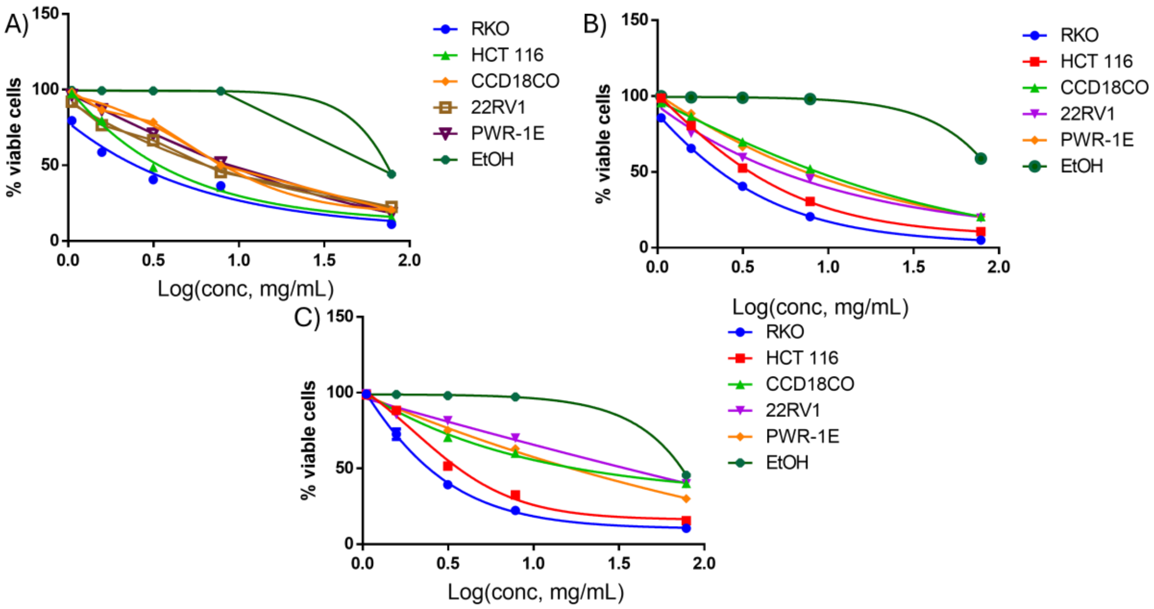

Supplementary Figure S1. The anti-proliferative effect of *Psidium guajava* measured at A) 24H after treatment with 78.75, 7.875 , 3.15 , 1.575 , and 1.05 mg/mL, B) 48H after treatment with 78.75 , 7.875 , 3.15 , 1.575 , and 1.05 mg/mL, and C) 72H after treatment with 78.75 , 7.875 , 3.15 , 1.575 , and 1.05 mg/mL. Log (conc, mg/mL) = log (concentration of bioactive compounds, mg/mL) (mean ± SD, n=3)
